# *Withania somnifera* Water Extract as a Potential Candidate for Differentiation Based Therapy of Human Neuroblastomas

**DOI:** 10.1371/journal.pone.0055316

**Published:** 2013-01-31

**Authors:** Hardeep Kataria, Renu Wadhwa, Sunil C. Kaul, Gurcharan Kaur

**Affiliations:** 1 Department of Biotechnology, Guru Nanak Dev University, Amritsar, India; 2 National Institute of Advanced Industrial Science and Technology, Tsukuba, Japan; Ospedale Pediatrico Bambino Gesu', Italy

## Abstract

Neuroblastoma is an aggressive childhood disease of the sympathetic nervous system. Treatments are often ineffective and have serious side effects. Conventional therapy of neuroblastoma includes the differentiation agents. Unlike chemo-radiotherapy, differentiation therapy shows minimal side effects on normal cells, because normal non-malignant cells are already differentiated. Keeping in view the limited toxicity of *Withania somnifera* (Ashwagandha), the current study was aimed to investigate the efficacy of Ashwagandha water extract (ASH-WEX) for anti-proliferative potential in neuroblastoma and its underlying signalling mechanisms. ASH-WEX significantly reduced cell proliferation and induced cell differentiation as indicated by morphological changes and NF200 expression in human IMR-32 neuroblastoma cells. The induction of differentiation was accompanied by HSP70 and mortalin induction as well as pancytoplasmic translocation of the mortalin in ASH-WEX treated cells. Furthermore, the ASH-WEX treatment lead to induction of neural cell adhesion molecule (NCAM) expression and reduction in its polysialylation, thus elucidating its anti-migratory potential, which was also supported by downregulation of MMP 2 and 9 activity. ASH-WEX treatment led to cell cycle arrest at G0/G1 phase and increase in early apoptotic population. Modulation of cell cycle marker Cyclin D1, anti-apoptotic marker bcl-xl and Akt-P provide evidence that ASH-WEX may prove to be a promising phytotherapeutic intervention in neuroblatoma related malignancies.

## Introduction

Neuroblastoma (NB) is the most common extra-cranial pediatric tumor that is derived from neural crest precursors. It is often malignant and undifferentiated in nature and retains the capability to differentiate into a variety of cells, including neurons, melanocytes and schwann cells. Since NB frequently has heterogeneous neoplastic populations which are highly variable in their state of differentiation, it has been predicted that failure of the neural crest cells to fully differentiate causes the development of neuroblastoma [1]. These have been extensively studied as a neoplastic model and to develop differentiation based chemo-therapies [1], [2] that are often complicated by the requirement of high doses and their cytotoxicity. Amongst others, retinoid-based differentiation and maintenance therapy has relatively increased survival rate for NB patients, however, there is still a significantly considerable number of patients showing relapse and deteriorated phases of NB [3], [4], [5]. Therefore, new treatment strategies are necessary to overcome existing shortcomings of conventional therapies.

*Withania somnifera* commonly known as Ashwagandha/Indian ginseng/Winter cherry is one of the most esteemed medicinal plants used in Ayurveda (Indian traditional medicine system) for over 3000 years. It has been used for all human age groups and no side effects have been reported so far [6]. Ashwagandha extracts as well as its different isolated bioactive constituents have been demonstrated to possess beneficial adaptogenic, anticancer, anti-convulsant, immunomodulatory, antioxidative and neurological effects. Several bioactive alkaloids and steroidal-lactone based phytochemicals, e.g. Ashwagandhine, Cuscohygrine, Isopelletierine, Anaferine, Anhygrine, Tropine, Sitoindosides (Saponins) and the diversely functional Withanolides, Withanamides, and Glycowithanolides have been isolated from different parts of this plant [7], [8], [9]. Its increasing therapeutic benefits continuously attract the attention of pharmacologists for biomedical investigations on plant extracts and isolated phytochemicals [10], [11], [12]. Recently reports from our lab and others have revealed the role of Ashwagandha in neuroprotection [13], [14].

Neuronal-differentiation and neuro-oncogenesis are multifactorial processes and are known to be influenced by multiple cell-signaling pathways including cytoskeleton and cell adhesion, stress and growth factor responses. Neurofilaments (NFs) are major cytoskeletal components of neurons and are composed mainly of three different polypeptide subunits: NF-L (68 kDa); NF-M (160 kDa); and NF-H (200 kDa). NF200 is expressed mainly in differentiated neurons [15], [16]; its phosphorylated form is extensively used as axonal marker. The expression pattern of many heat shock proteins appears to be closely linked in early mammalian development to critical differentiation and proliferation stages [17], [18]. Some stress chaperones, such as mortalin perform multiple functions relevant to multiple stress reponse, cell survival and differentiation [19], [20], [21].

The neural cell adhesion molecule (NCAM) exhibits high structural diversity and it has been implicated in a multitude of cellular functions, not only during neural development and plasticity but also in oncogenesis [22], [23]. The most prominent and unique posttranslational modification of NCAM is addition of polysialic acid (PSA), to the fifth immunoglobulin like domain of NCAM [24]. Despite the abundant evidence that polysialic acid is critically involved in neural development and tumor malignancy, its mode of action on the cellular level is still unclear [25]. Akt (protein kinase B), is an important regulator for multiple biological processes, including metabolism, cell size, apoptosis, and cell cycle progression [26]. Cyclin D1, a proto-oncogene and an important regulator of G1 to S-phase transition and bcl-xl (an important member of bcl-2 anti-apoptotic family of proteins) have also been shown to regulate neuronal differentiation [27], [28].

In an effort to expand strategies for targeting neuroblastoma cells, the current study explored the potential anti-cancer activity of ASH-WEX on neuroblastoma cells. In the present study, we demonstrate that ASH-WEX is a potent anti-proliferative and proapoptotic agent of neuroblastoma cells *in vitro*. These effects are associated with an increased expression of Neurofilament (NF200), HSP70, mortalin, NCAM and a concomitant inhibition of PSA-NCAM expression. We also demonstrate that ASH-WEX inhibits the MMP-2 and MMP-9 activity and cell migration and to further ascertain the underlying signalling pathways the expression of cell cycle marker Cyclin D1, anti-apoptotic marker bcl-xl and Akt-P were studied. Cell cycle analysis and Annexin V-FITC/PI staining were done to ascertain the cell state after ASH-WEX treatment. As such, these findings identify ASH-WEX as a potential therapeutic agent for the treatment of high grade neuroblastomas.

## Materials and Methods

### Ashwagandha Water Extract Preparation (ASH-WEX)

ASH-WEX was prepared as reported earlier [29]. Briefly, 10 g of dry leaf powder was suspended in 100 ml of distilled water and stirred overnight at 45±5°C, followed by filtration under sterile conditions. The filtrate thus obtained was treated as 100% ASH-WEX.

### Cell Culture and Treatments

IMR-32, SH-SY5Y and Neuro-2a cell lines were obtained from National Centre for Cell Sciences (Pune, India) and TGW cell line was obtained from the Health Science Research Resources Bank (Osaka, Japan). IMR-32, TGW and Neuro-2a neuroblastoma cell line were maintained on DMEM supplemented with 1X PSN (Invitrogen), 10% FBS (Biological Industries) at 37°C and humid environment containing 5% CO_2_. SH-SY5Y cells were maintained on DMEM/Ham’s F-12 (1∶1) nutrient mixture supplemented with 10% FBS. Cultures at 30–40% confluency were treated with ASH-WEX (0.01– 1.0% diluted in medium) for 72 h. The control cultures were given a medium change alone. For comparisons a standard differentiation inducing agent Retinoic acid (RA) was included in the study. RA (10 µM) treatments were given in the presence of 10% FBS similar to ASH-WEX treatment groups. To ascertain the effect of the vehicle, DMSO in the RA treated group, DMSO (1 µl/ml) treated cells were studied along with the control (untreated) IMR-32 cells for 72hrs (Figure S1).

### Cell Proliferation Assay and Morphological Study

ASH-WEX was tested for anti-proliferative activity on IMR-32, TGW, SH-SY5Y and Neuro-2a neuroblastoma cells using the 3-(4, 5-dimethylthiazol-2-yl)-2, 5-diphenyltetrazolium bromide (MTT) test. Morphological changes in neuroblastoma cells treated with different concentrations of ASH-WEX were examined by phase contrast microscopy.

### Immunostaining

The control and treated IMR-32 neuroblastoma cells were fixed with paraformaldehyde followed by permeabilization with 0.3% PBST. After blocking with 5% normal goat serum (NGS), the coverslips were incubated with anti NF-200 (1∶500, Sigma), anti-HSP70 (1∶1000, Sigma), anti-NCAM (1∶500, Sigma), anti-PSA-NCAM (1∶200, Abcys), anti-Cyclin D1 (1∶500, Sigma), anti-bcl-xl (1∶100, Sigma) and anti-Akt-P (1∶200, Sigma) diluted in 0.1% PBST, for 24h at 4°C in humid chamber. Secondary antibody (goat anti-mouse/IgG/IgM Alexa Fluor conjugated from Invitrogen) was applied for 2h at room temperature. Cells were then mounted with anti-fading reagent and observed. Each experiment was carried out in duplicate and repeated thrice. Images were captured using Cool Snap CCD camera and the pictures were analyzed using Image pro-plus software version 4.5.1 from the media cybernetics.

### Western Blotting

Cells were grown and treated in 90 mm petri-plates were harvested with PBS-EDTA (1 mM). Cell pellet was homogenized in NP-40 lysis buffer (50 mM Tris, 150 mM NaCl, 0.5% Sodium deoxycholate, 0.1% SDS, 1.0% NP-40) and protein content in the supernatant was determined by the Bradford method. Protein lysate (20–30 µg) was resolved on 10% SDS-PAGE followed by blot transfer onto a PVDF membrane (Hybond-P) using the semidry Novablot system (Amersham Pharmacia). Subsequently, membranes were be probed with mouse anti-NF 200 (1∶2000), anti-HSP70 (1∶5000), anti-NCAM (1∶2000), anti-PSA (1∶1000), anti-Cyclin D1 (1∶2500), anti-bcl-xl (1∶1000) and anti-Akt-P (1∶2000, Sigma). Immunoreactive bands were visualized using ECL Plus western blot detection system (Amersham Biosciences). In order to account for potential variations in protein estimation and sample loading, expression of each protein was compared to that of α-tubulin in each sample after stripping the blot. Each experiment was repeated thrice.

### mRNA Expression by Semiquantitative RT-PCR

Total RNA was extracted from the cells by the TRI reagent (Sigma) according to manufacturer’s instruction. Equal amount of RNA was used for cDNA synthesis. cDNA was synthesized in 20 µl reactions containing 200 units M-MLV reverse transcriptase, 4 µl 5×first strand buffer, 2 µl DTT (0.1 M) (Invitrogen), 5 µg of total RNA, 1 mM each of dNTPs (Amersham), 20U of ribonuclease inhibitor (Sigma), and 250 ng pd(N)_6_ random hexamers (MBI, Fermentas). 2 µl of cDNA was amplified in a 50 µl PCR reaction mixture containing 2U Taq polymerase, 5 µl 10×PCR buffer, 1.5 µl of 50 mM MgCl_2_ (Sigma), 1 µl of 10 mM dNTP mix (Amersham), and 20 pM respective primers (Table 1). Cycling conditions comprised of an initial denaturation of 3 min at 94°C followed by 35 cycles of amplification (at 94°C for 40 sec, 55°C for 45 sec and 72°C for 1 min) and final elongation step at 72°C for 10 min. To control the PCR reaction components and the integrity of the RNA, 2 µl of each cDNA sample was amplified separately for β-actin specific primer. Each experiment was repeated thrice.

**Table 1:** Primer sequences used for semi-quantitative RT-PCR.

No.	mRNA	Primer Sequence	Expected product size
1.	NF200	F 5′CAAGGAACCCAGCAAACCA3’ R 5′GGCCTCTGTCTTGGGTTTCTC3’	106 bp
2.	HSP70	F 5′GAGTTCAAGCGCAAACACAA3’ R 5′CTCAGACTTGTCGCCAATGA3’	428 bp
3.	Mortalin	F 5′CAGTCTTCTGGTGGATTAAG 3′ R 5′ATTAGCACCGTCACGTAACACCTC 3′	420 bp
4.	NCAM	F 5′TGAGGGTACTTACCGCTGTG3’ R 5′GTTGCTGGCAGTGCACATGT3’	651 bp
5.	PST	F 5′TAAGGTGCAATCTAGCTCCTGTGGTGG3’ R 5′GCATCCTGTGAGGACTGGCGTTGGAAA3’	474 bp
6.	MMP-2	F 5′GGCTGGTCAGTGGCTTGGGGTA3’ R 5′AGATCTTCTTCTTCAAGGACCGGTT3’	200 bp
7.	MMP-9	F 5′TGTACCGCTATGGTTACAC3’ R 5′CCGCGACACCAAACTGGA3’	357 bp
8.	Cyclin D1	F 5′ATGGAACACCAGCTCCTGTGCTGC3’ R 5′TCAGATGTCCACGTCCCGCACGT3’	888 bp
9.	Bcl-xl	F 5′AGGATACAGCTGGAGTCAG3’ R 5′TCTCCTTGTCTACGCTTTCC3’	417 bp
10.	Akt	F 5′ACGACCGCCTCTGCTTTG3’ R 5′ACACGCGCTCACGAGACA3’	101 bp
11.	β-actin	F 5′TCACCCACACTGTGCCCATCTACGA3’ R 5′CAGCGGAACCGCTCATTGCCAATGG3’	285 bp

### Wound Scratch Assay

In order to investigate IMR-32 cell migration capability after ASH-WEX treatment, cells were grown to confluent monolayer. The monolayer was wounded by scratching the surface with a sterile needle (22 gauge). The initial wounding and the movement of the cells in the scratched area was photographically monitored for 24 h after the treatment with the ASH-WEX extract. The assay was done in duplicate and repeated thrice.

### Matrix Metalloproteinases (MMPs) Zymography

Samples of supernatant medium conditioned by cell culture under different experimental conditions were separated on a 10% SDS-PAGE containing 0.1% gelatin. After electrophoresis, gels were washed with 2.5% Triton X-100 (in 50 mM Tris-HCl) for 30 min to remove SDS, followed by incubating the gel in zymogram developing buffer (Invitrogen) at 37°C for 48 hrs. Gels were subsequently stained with Coomassie brilliant blue and destained in buffer containing 50% methanol and 10% acetic acid (v/v), and the location of gelatinolytic activity was detected as clear bands. Samples from three different experiments were analysed for quantitative analysis.

### Cell Cycle Analysis Using Propidium Iodide

Cells were plated at 2 × 10^5^ cells/dish in 10 cm diameter dishes, and then grown either in the presence or absence of ASH-WEX and RA. After 72 hrs of treatment, the cells were harvested from dishes by collecting trypsinized cells together with floating cells in the medium. For each condition, a volume of the cell suspension corresponding to 2 × 10^6^ cells was centrifuged and the resultant cell pellet was resuspended in ice-cold PBS (1.0 ml). Cells were fixed in ice-cold 70% ethanol and stained with propidium iodide. FACS analysis was performed using a BD Accuri C6 flow cytometer (BD Biosciences Immunocytometry Systems, San Jose, CA). DNA content histograms and cell cycle phase distributions were modelled from at least 20,000 single events by excluding cell aggregates based on scatter plots of fluorescence pulse area versus fluorescence pulse width using FCS Express 4 flow research edition software (De novo software). The experiments were repeated thrice for further analysis.

### Annexin-FITC Apoptosis Assay

To determine the extent of apoptotic and necrotic cell death, cells were stained with annexin V conjugated with FITC and PI using the Annexin V-FITC Apoptosis Detection Kit (Miltneyi Biotech), according to the manufacturer’s protocols. Annexin V has a high affinity for phosphatidylserine exposed on the outer membrane of apoptotic cells, while PI is transported to late-stage apoptotic/necrotic cells with disrupted cell membranes. The cells from control and treated groups were trypsinized, washed with PBS, and resuspended in 1 ml of annexin V binding buffer (1×) with addition of 10 µl annexin V-FITC. Following incubation (for 15 min in the dark at room temperature) and centrifugation (5 min, 300×g), 500 µl of annexin V binding buffer and 5 µl of PI were added to the cell pellet and incubated for further 5 min in the same conditions. Then, viable (annexin V-, PI-negative), early apoptotic (annexin V-positive, PInegative), late apoptotic (annexin V-, PI-positive) and necrotic (annexin V-negative, PI-positive) cells were detected by flow cytometry (Accuri C6 flow cytometer; Becton–Dickinson) and quantified from three different experiments by FCS Express 4 flow research edition software (De novo software).

### Data Analysis

The captured images were analyzed using Image Pro-Plus software version 4.5.1 from Media Cybernetics. The extent of immunoreactivity was quantified by the overall density of their respective immunoreactivity each in 5–6 randomly selected fields on each image using the count/size command of the Image Pro-Plus software. 15 different images were used from three different experiments and the data were averaged and expressed as percentage with respect to control.

### Statistical Analysis

Values are expressed as mean ± SEM. The SigmaStat for Windows (version 3.5) was adopted to analyse the results by Student’s *t*-test in order to determine the significance of the means. Values of p<0.05 were considered as statistically significant.

## Results

### ASH-WEX Exerted Antiproliferative Effects and Induced Differentiated Morphology

**Figure 1:**
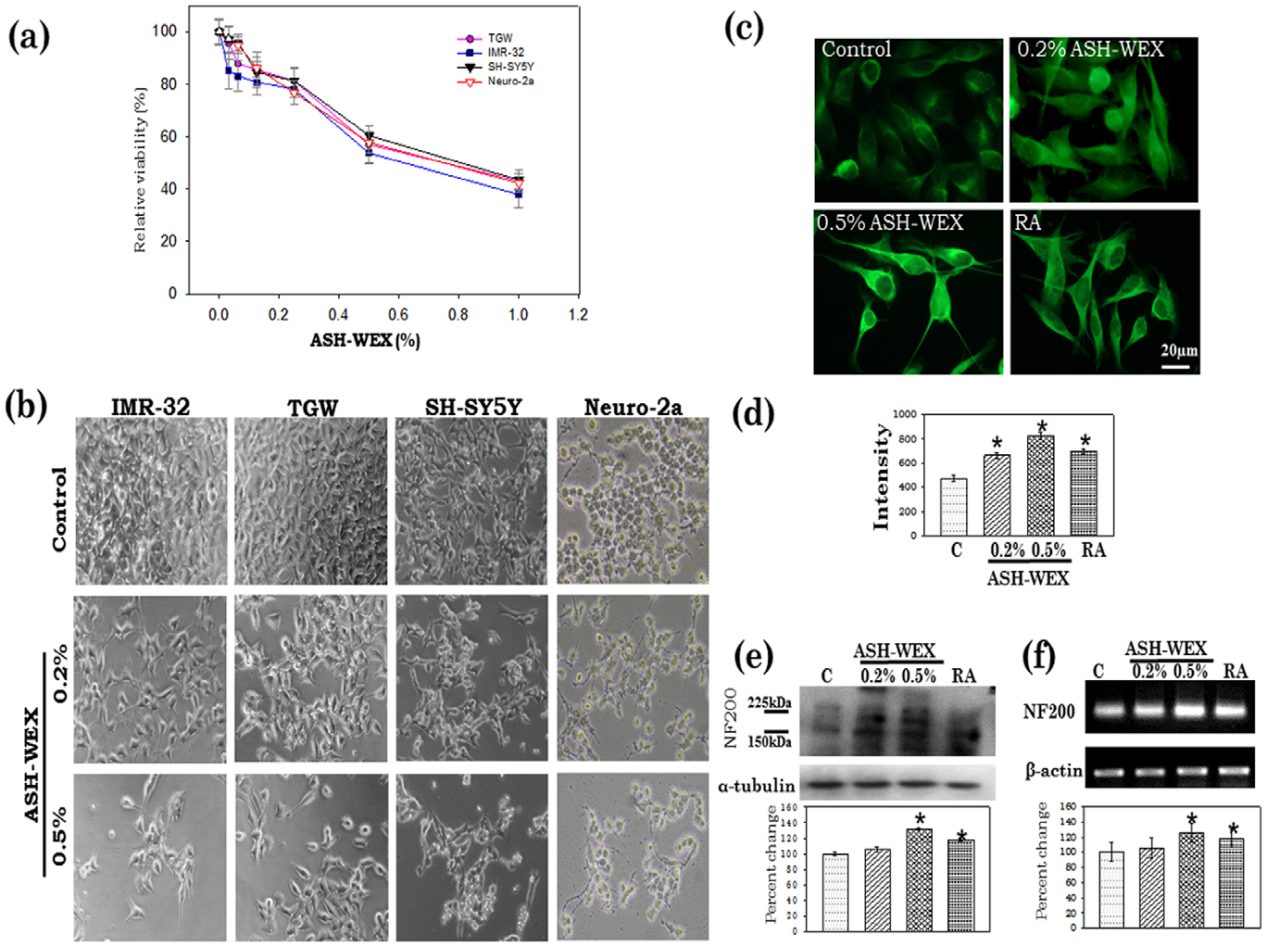
Growth curve inhibition as assessed by MTT assay in IMR-32, TGW, SH-SY5Y and Neuro-2a cells (a). Data are representative of three different experiments done in triplicates and expressed as mean ± S.E.M. (b) Phase contrast images of IMR-32, TGW, SH-SY5Y and Neuro-2a neurolastoma cells treated with 0.0% (Control), 0.2% and 0.5% ASH-WEX. (c) NF200 expression in response to ASH-WEX treatment in control, ASH-WEX (0.2% and 0.5%) and RA treated IMR-32 cultures. The relative intensity measurement of immunofluorescence is shown (d). (e) Representative Western blot hybridization signals for NF200 from control and test samples. (f) Representative RT-PCR results for NF200 mRNA in control and treated cells and their relative densitometry analysis represented by histograms. “*” represents the statistical significant (p<0.05) difference between control and ASH-WEX treated groups.

Human neuroblastoma cell lines IMR-32, TGW, SH-SY5Y and mouse neuroblastoma cell line Neuro-2a cells were cultured in the presence of different concentrations of ASH-WEX. The extract was cytotoxic (Fig. 1a) at concentrations higher than 0.5%. At lower concentrations (0.5% and less), cells appeared to be growth arrested with extended neurite like projections which appeared to be similar to the differentiated cells. The antiproliferative activity of ASH-WEX cell was further investigated by MTT assay (Fig. 1a). ASH-WEX was able to reduce the proliferation rate in all the four neuroblastoma cell lines. Based on the MTT data, we selected 0.2% and 0.5% ASH-WEX concentrations for testing its differentiation inducing potential and human neuroblastoma cell line IMR-32 was selected for further studies.

IMR-32 neuroblastoma cells when treated with 0.2% and 0.5% of ASH-WEX, showed significant morphological changes as compared to control cells (Fig. 1b). The treated cells showed extended and multiple projections. To confirm the induction of differentiation, the expression of mature neuronal marker, NF200 was examined. Furthermore 10 µM RA treated cells were taken as positive control to compare the differentiation status. ASH-WEX treatment lead to increased expression of NF200 (Fig. 1c); Comparison of the NF200 expression in ASH-WEX and RA treated cells revealed that ASH-WEX was a strong inducer of neuronal differentiation (Fig. 1d)). Increase in NF200 expression in 0.2% and 0.5% treated cells was also confirmed by Western blotting and RT-PCR to ascertain the changes at both translational and transcriptional levels (Fig. 1e,f).

### ASH-WEX Induced HSPs and Senescence in Neuroblastoma

**Figure 2:**
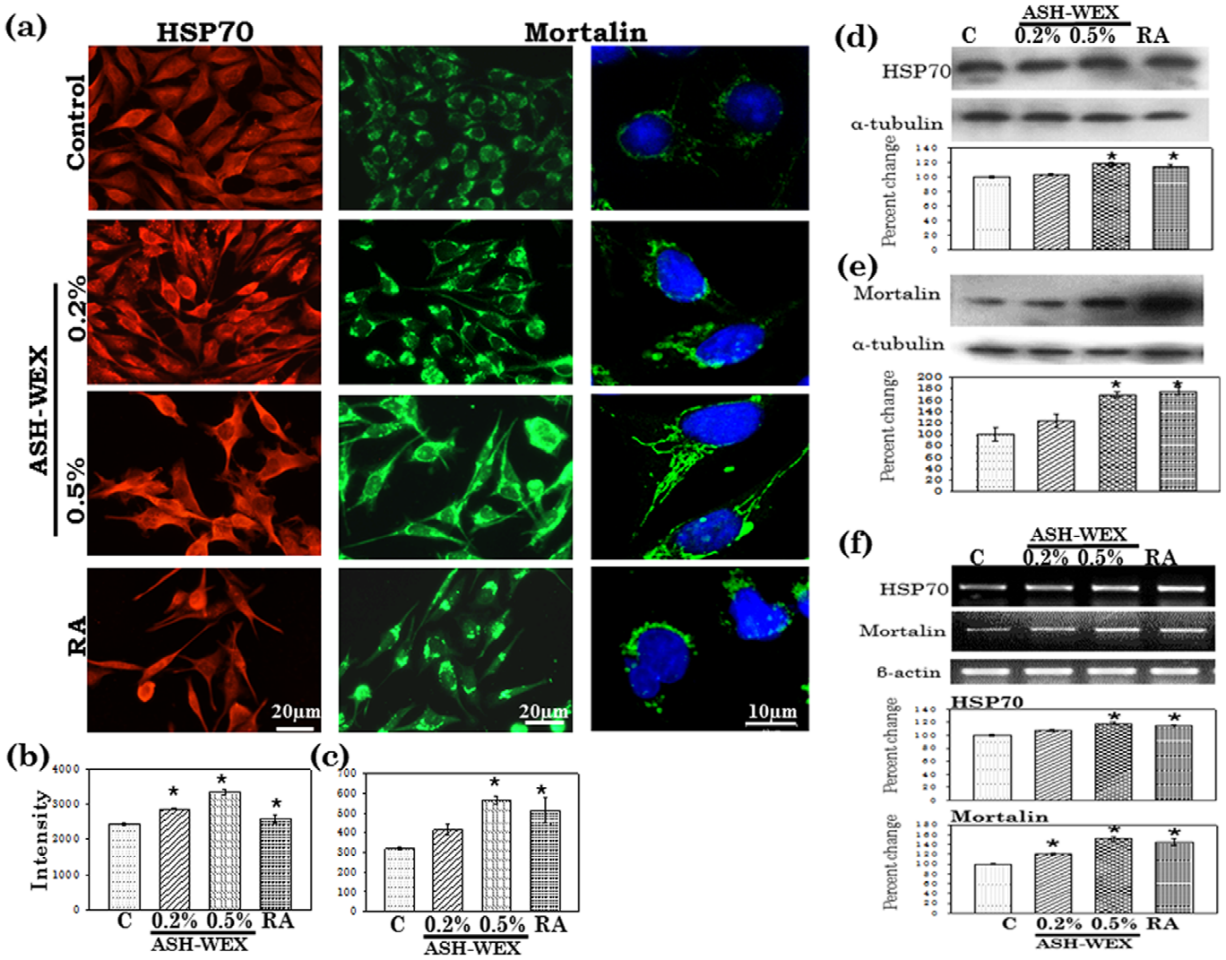
HSP70 and Mortalin expression in response to ASH-WEX treatment in control, ASH-WEX (0.2% and 0.5%) and RA treated IMR-32 cultures (a). The relative intensity measurement of immunofluorescence is shown as histogram for HSP70 (b) and Mortalin (c). Representative Western blot hybridization signals for HSP70 (d) and Mortalin (e) from control and test samples and their relative intensity. Representative RT-PCR results for HSP70 and Mortalin mRNA in control and treated cells and their relative densitometry analysis represented by histograms. “*” represents the statistical significant (p<0.05) difference between control and ASH-WEX treated groups.

HSP70 expression was increased with 0.5% ASH-WEX treatments and the increase in HSP70 immunoreactivity was comparable to that of 10 µM RA treated group (Fig. 2a,b). Western blot and RT-PCR results further supported these observations as there was significant increase in HSP70 expression (Fig. 2d,f). We further examined the ASH-WEX and RA treated cells for the induction of senescence using mortalin staining as a marker. It was observed that the ASH-WEX treated cells showed pancytoplasmic staining of mortalin in 80–90% of the cells indicating the senescent status as compared to perinuclear staining in the control undifferentiated cells (Fig. 2a). Furthermore, we found that mortalin was also localized into the nucleus of 0.5% ASH-WEX treated cells. In contrast, RA treated group showed a unipolar expression of mortalin. Mortalin staining intensity was significantly higher in ASH-WEX treated cells (Fig. 2c) which was also confirmed by Western blotting results and further mRNA expression changes are supported by RT-PCR data (Fig. 2d,f).

### ASH-WEX Modulated Cell Cycle, Apoptotic and Survival Markers

**Figure 3:**
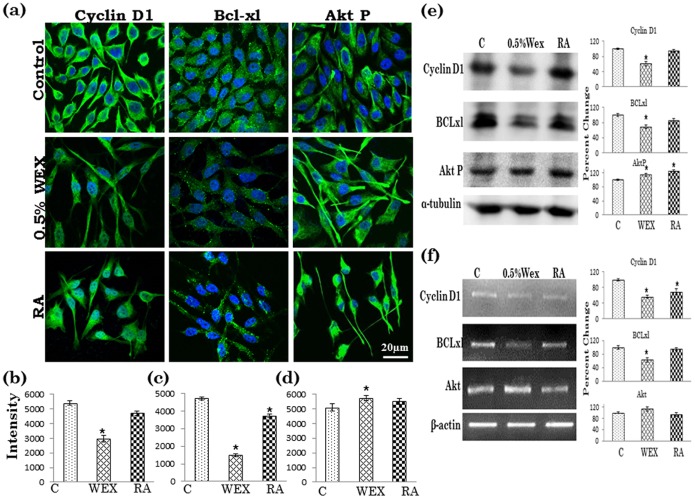
Immunofluorescence detection of Cyclin D1, bcl-xl and Akt-P is shown in control, 0.2% and 0.5% ASH-WEX, RA treated IMR-32cells (a). Relative intensity measurement of immunofluorescence is shown as histograms for Cyclin D1 (b), bcl-xl (c) and Akt-P (d). Representative Western blot hybridization signals for Cyclin D1, bcl-xl and Akt-P from control and test samples (0.5% ASH-WEX and RA treated cells) (e). mRNA expression analysis for Cyclin D1, bcl-xl and Akt-P was done and densometery results for intensity analysis are represented as histogram (f). “*” represents the statistical significant (p<0.05) difference between control and ASH-WEX treated groups.

To further look into the possible signalling pathways associated with antiproliferative, antimigratory and differentiation inducing potential of ASH-WEX, the expression of Cyclin D1, bcl-xl and Akt-P were examined. Only 0.5% ASH-WEX treatment group was further used for these studies as higher concentration of ASH-WEX showed more promising results as compared to 0.2% ASH-WEX group. 0.5% ASH-WEX treatment lead to significant decrease in expression of Cyclin D1 as assessed by quantitative analysis of intensity of immunocytofluorescence (Fig. 3a,b). The expression was significantly low at both the translational as well as transcriptional level as assessed by Western blotting and RT-PCR, respectively (Fig. 3e,f) and more pronounced changes were observed in ASH-WEX treated cells as compared to RA treated group.

Anti-apoptotic marker bcl-xl was significantly decreased upon 0.5% ASH-WEX treatement as assessed by immunocytofluorecence and expression was significantly lower than the RA treated group (Fig. 3a,c). Western blotting and RT-PCR results also showed significant downregulation of bcl-xl expression upon ASH-WEX treatment (Fig. 3e,f). Phosphorylation of Akt appeared to be induced upon treatment with both ASH-WEX and RA. Furthermore, it was localized more into the neurite like projections of the cells in the treated cells (Fig. 3a). The increase in Akt-P expression was not statistically significant in the ASH-WEX group as shown by Western blotting results (Fig. 3e). The mRNA expression of Akt was also increased, although not significantly, in the ASH-WEX treated group (Fig. 3f).

### ASH-WEX Leads to G0/G1 Cell Cycle Arrest

**Figure 4:**
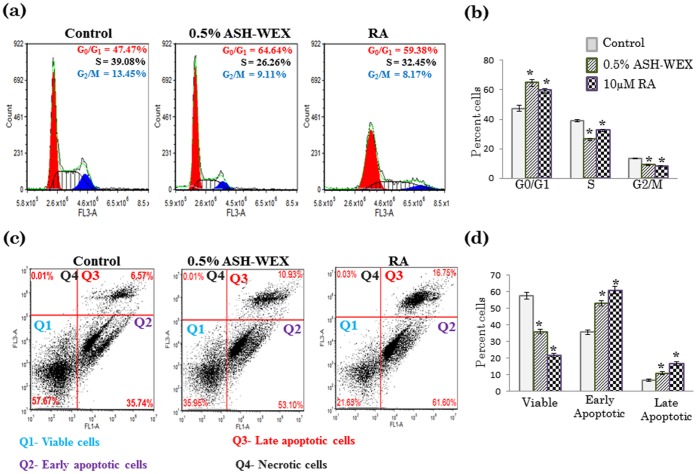
ASH-WEX affects the distribution of events in the IMR-32 cell cycle. IMR-32 cells were treated with 0.5% ASH-WEX and RA for 72 h (a). The evaluation of cell cycle progression was done by DNA staining by propidium iodide. The figure shows representative FACS profiles of the distribution of cells in G0/G1, S, and G2/M phases as analysed by FCS software. (b) Histogram represents percentage distribution of the cells in different phases (G0/G1, S, and G2/M) after ASH-WEX treatment as compared to control. (c) Flow cytometric examination of apoptosis, necrosis and cell viability-the Annexin V/PI assay. Diagrams show four subgroups of cells. Viable (Q1, annexin V-, PI-), early apoptotic (Q2, annexin V+, PI-), late apoptotic (Q3, annexin V+, PI+) and necrotic/damaged (Q4, annexin V-, PI+) are represented in different quadrants. (d) Histogram represents percentage distribution of the cells in different quadrants. “*” represents the statistical significant (p<0.05) difference between control and ASH-WEX treated groups.

Since it has been reported that cell cycle arrest at the G0/G1 or G2/M boundaries, as well as cytokinetic block may be indicative of senescence-like alterations and followed by cell death events, we analyzed cell cycle distribution in IMR-32 cells in our experimental conditions. As regards the G2/M and S phases of the cycle, there was a significant decrease in the median percentage of cells with DNA content corresponding to these phases after treatment with 0.5% ASH-WEX and RA, in comparison with the population of control cells (Fig. 4a). There were only 26.26% cells in S phase in ASH-WEX treated group as compared to 39.08% in control and 32.45% in RA treated groups. Concurrently, a significant increase in the median percentage of cells classified as G0/G1, according to their DNA content, has been observed as a consequence of exposure to 0.5% ASH-WEX and RA. ASH-WEX treated group showed highest percentage of cells (64.64%) in G0/G1 phase as compared to 47.47% in control and 59.38% in RA treated groups (Fig. 4b).

To further verify these observations and to resolve the question if the above-described fluctuations in the percentages of cells between cell cycle phases were related to G0/G1 arrest and consequently differentiation, or rather to an elevated rate of cell death in G0/G1, Annexin V-FITC and PI staining was performed (Fig. 4c). With 0.5% ASH-WEX treatment, the median values for annexin V-positive/PI-negative (early apoptotic), annexin V-positive/PI-positive (late apoptotic), annexin V-negative/PI-positive (necrotic) cells were 53.10, 10.93 and 0.01%, respectively which were significantly higher than the control group, indicating a shift toward late apoptosis. RA treatment group further showed an increase in number of early apoptotic (61.60%) and late apoptotic (16.75%) cells, as compared to the control (Fig. 4c,d).

### ASH-WEX Induced Anti-migratory Properties in IMR-32 neuroblastoma Cells

**Figure 5:**
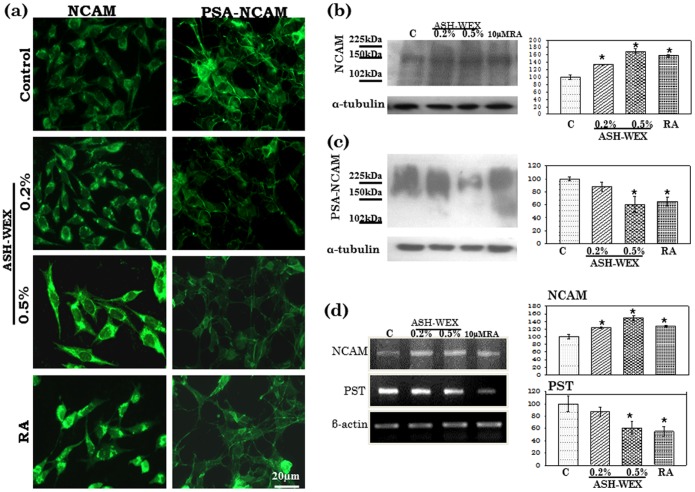
NCAM and PSA-NCAM expression in response to ASH-WEX treatment in control, ASH-WEX (0.2% and 0.5%) and RA treated IMR-32 cultures (a). Representative Western blot hybridization signals for NCAM (b) and PSA-NCAM (c). Representative RT-PCR results for NCAM and PST mRNA in control and treated cells and their relative densitometry analysis represented by histograms. “*” represents the statistical significant (p<0.05) difference between control and ASH-WEX treated groups.

**Figure 6:**
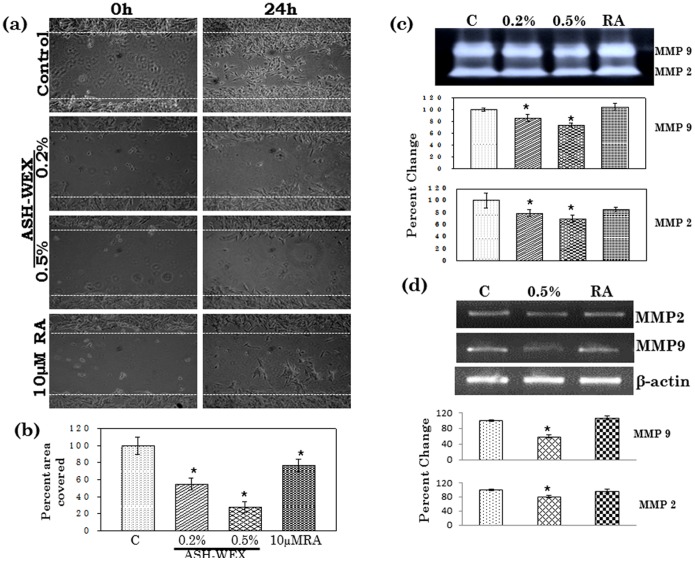
Representative phase contrast images of control, 0.2% or 0,5% ASH-WEX and RA treated cells, in which motility was analyzed by Wound-scratch test (a). Images show the starting (0 h after scratch) and the end (24 h after scratch) point of the analysis. (b) Graph shows that the rate of IMR-32 migration in response to ASH-WEX treatment in comparison to untreated cells. Data are obtained from a set of scratch test analysis (N  = 3) and are expressed as means ± standard error. Representative MMP zymogram from control and treated samples and their densometery analysis is represented as histogram (c). mRNA expression for MMP2 and MMP9 was analyzed by RT-PCR. Relative percentage expression was expressed as histogram (d). “*” represents the statistical significant (p<0.05) difference between control and ASH-WEX treated groups.

Expression of plasticity markers such as neural cell adhesion molecule and its polysialylated form (NCAM and PSA-NCAM) was studied in IMR-32 cells to investigate their adhesion and migratory characteristics with and without the treatment with ASH-WEX. The protein and mRNA expression of NCAM was significantly increased in cells treated with 0.5% ASH-WEX as detected by immunostaining, Western blotting and RT-PCR (Fig. 5a,b,d) which was even higher than the RA treated group. Polysialylation of NCAM was significantly reduced upon treatment with ASH-WEX as the expression of PSA-NCAM was minimal in 0.5% ASH-WEX treated group as depicted by immunostaining and Western blotting. In the control group 80–90% cells expressed PSA-NCAM which was downregulated after ASH-WEX treatment. Only 5–10% cells seemed to be stained positive for PSA-NCAM in 0.5% ASH-WEX (Fig. 5a). Expression of polysialyltransferase (PST) was evaluated using RT-PCR in the ASH-WEX and RA treated cells. There was significant decrease in the expression of PST when treated with ASH-WEX as compared to control group (Fig. 5c,d). To evaluate the motility of IMR-32 cells, wound-healing assay was performed with and without adding ASH-WEX and 10 µM RA in these groups. As shown in Fig. 6a, untreated IMR-32 cells were able to invade the scratched area that was fully re-colonized by 24 hr. 0.2% and 0.5% water extract treatment significantly reduced the migration rate of the IMR-32 neuroblastoma cells. Very few cells were seen in the scratched area in the 0.5% treatment group as compared to 10 µM RA treated group after 24 hrs of treatment. Quantitative analysis also indicated a significant decrease (about 27 to 55%) of the cell migration rate following ASH-WEX treatment which was around 76% in RA treated group (Fig. 6b). Further gelatin zymography was performed to assess the activity of MMP-2 and MMP-9 matrix metalloproteinases to co-relate with the anti -migratory properties of ASH-WEX. It was found that MMP-2 and MMP-9 activities were reduced significantly in the 0.5% treated group as compared to control and 10 µM RA group. The MMP-2 activity was apparently more decreased out of the two MMPs analysed (Fig. 6c) and the change was statistically significant. In contrast, RA treatment group showed slight induction of MMP2 activity. Expression of MMP-2 and MMP-9 was analysed at mRNA level and 0.5% ASH-WEX treatment lead to significant decrease in their expression as compared to control group (Fig. 6d).

## Discussion

Differentiation therapy focuses on the development and use of specific agents designed to selectively engage the process of terminal differentiation, leading to the eventual elimination of tumorigenic cells and rebalance of normal cellular homeostasis. In the present study, the differentiation inducing potential of Ashwagandha was evaluated for its activity against neuroblastoma cell lines. RA is a potent regulator of neuroblastoma cell differentiation [30] and used in number of cancer differentiation based therapeutics. RA and its derivatives activate retinoic acid receptors and retinoid X receptor (RAR-RXR) complexes and induce neural differentiation of NSCs [31]. We took 10 µM RA as a positive control for differentiation, to compare the potential of ASH-WEX with conventional differentiation inducing agent RA. ASH-WEX was able to inhibit the cell proliferation in a dose dependent manner in the IMR-32, TGW, SH-SY5Y and Neuro-2a neuroblastoma cell lines. Further it was observed to induce differentiated morphology in these cell lines at 0.5% ASH-WEX dose with neurite like projections. These observations were further confirmed by NF200 expression which showed significant increase in ASH-WEX treated cells which was more pronounced as compared to RA-treated group. NF200 and its phosphorylated is a marker of differentiation of neuronal cells [16]. In an earlier study, the constituents of alcoholic extract of Ashwagandha have been shown to elevate level of NF200 in neuroblastoma cells along with neurite outgrowth [32], [33]. Consistent with these reports the increase in expression of NF200 in the present study could be attributed to differentiation inducing activity of ASH-WEX in the neuroblastoma cells.

The differentiation state is accompanied by the formation of dendrites and axonal processes and requires an increase in protein transport. In the present study, HSP70 level was found to be elevated when treated with ASH-WEX which was maximum in the RA treated group. HSP70 is an essential ATP-dependent uncoating enzyme and its induction is important in the neuronal differentiation and neurite extension [34], [35]. Another natural molecule, Celastrol has been shown to induce HSP70 both in undifferentiated neuroblastoma cells [36]. Thus the increase in HSP70 expression after ASH-WEX and RA treatment may support their differentiation inducing activity the IMR-32 cells.

The expression of mortalin in the ASH-WEX treated cells shifted from juxtanuclear to pancytoplasmic pattern. Furthermore, the expression of mortalin was significantly upregulated upon 0.5% ASH-WEX treatment. Earlier studies have demonstrated that immortal cells display a perinuclear distributon of mortalin, whereas, the normal mortal cells exhibit a pancytoplasmic expression [37]. Also, mortalin carries the function of control of cell proliferation and differentiation [21], [38]. Its role in neuroblastoma cell differentiation has been established in another recent study as a favorable prognostic indicator of neuroblastoma differentiation [39]. Thus the pancytoplasmic distribution and enhanced expression of mortalin further suggests that ASH-WEX treatment may be inducing cellular senescence and also confirms the differentiation status of the IMR-32 cells in treated cells. These results are also supported by our earlier findings of Ashwagandha induced mortalin expression in glioma cells [29], [40]. Furthermore 0.5% ASH-WEX treated cells showed nuclear localization of mortalin which was apparently more than the RA treated group. Recent study has shown the association of mortalin with RARa and RXRa is remarkably increased in the nucleus and coincides with the RA-elicited growth arrest, concomitant with a tight correlation between RA-induced nuclear translocation of mortalin and RA triggered neuronal differentiation [21].

ASH-WEX treatment of IMR-32 cells resulted in downregulation of cyclin D1 expression at transcriptional as well as translational level. Genetic aberrations and over-expression of the Cyclin D1 gene have been reported for several human neoplasms and neuroblastomas [41], [42] and elevated expression of cyclin D1 is associated with high degree of malignancy and rapid cell proliferation [43]. Moreover, Cyclin D1 overexpression has been reported to prevent differentiation in neuroblastoma [44]. On the contrary, downregualtion/silencing of Cyclin D1 mRNA leads to neuronal outgrowth and differentiation [42]. Recently some studies have functionally linked neuronal differentiation to cell cycle regulation which frequently involves the G1 cell cycle entry point [28], [45], [46], [47]. The current study further investigated the phase population in the cell cycle after treatment with ASH-WEX. Analysis of neuroblastoma cells after ASH-WEX treatment revealed a strong differentiated phenotype. FACS analysis of these cells showed an increase of the G0/G1 fraction at 72 hours after treatment which was even better than RA treated cells under similar conditions. In concordance with the arrest of the cell cycle in G1 phase, ASH-WEX treatment resulted in a reduction in cyclin D1 protein levels, thus suggesting that neuroblasts differentiate towards a neuronal phenotype after inhibition of the G1 checkpoint. Apart from cell cycle regulation these G1 entry checkpoint regulators have been linked to other signal transduction routes. The involvement of Cyclin D1 in neuronal differentiation processes has been suggested by earlier studies [46], [48]. This is in line with the findings that growth signaling pathways determine differentiation patterns in non-malignant neuroblasts and influence the differentiation state of neuroblastoma [42]. These signal transduction routes most frequently involve the transcriptional regulation of Cyclin D1 and thus the effect on neuronal differentiation by these signal transduction routes could partly function through Cyclin D1 regulation [49]. Inhibition of the G1 regulating genes CDK4 or Cyclin D1 in neuroblastoma cell lines lead to the restoration of the G1 checkpoint and subsequent neuronal differentiation [50]. In our previous study we have established that Ashwagandha alcoholic extract causes cell cycle arrest at G2/M [40]. The difference could be due to different nature of extracts used and thereby different bioactive molecules and their mode of action. As cell cycle arrest is a prerequisite of differentiation, it is reasonable to relate the role of ASH-WEX in regulating cell cycle leading to G0/G1 cell cycle arrest with downregulation of cyclin D1 and consequent differentiation of the IMR-32 cells. Annexin V-FITC/PI staining study further supports this observation as there is increase in early apoptotic cell population which may be due to differentiation inducing ability of ASH-WEX thus ultimately leading to normal cell apoptotic pathway.

Most of the neuroblastoma cells, including IMR-32, are resistant to apoptosis and differentiation. Bcl-xl is widely expressed in neuroblastoma cells and inhibits chemotherapy-induced apoptosis [51]. Anti-apoptotic functions of bcl-xl are well known. Recent reports on curcumin, andrographolide, cranberry proanthocyanidines have established bcl-xl mediated pro-apoptotic properties of natural compounds [52], [53]. Therefore we evaluated whether ASH-WEX could influence bcl-xl expression in the IMR-32 cells. ASH-WEX lead to downregulation of bcl-xl both at transcriptional and translational level, which further supports pro-apoptotic potential of ashwagandha extracts in the cancerous cells [54]. Akt is another important cell signalling molecule involved in cell proliferation and survival. Akt-P expression was upregulated in the IMR-32 cells upon treatment with ASH-WEX. It is known that Akt is at a pivotal nodal point in the signaling pathway of almost all RTKs and is activated by the phosphoinositol-3-kinase. It has been reported earlier that in Neuro2a neuroblastoma cells, Akt showed increased phosphorylation after serum withdrawal leading to differentiation [55]. Also estrogen and mevastatin has been known to induce Akt mediated neurite outgrowth leading to differentiatiaon in neuroblastoma cells [56], [57]. In keeping with this proposal, Perez-Tenorio and co-workers showed P-Akt to strongly associate with a lower S-phase fraction [58]. In addition to its vital function in cell survival, a role for PI3K/Akt signalling has also been implicated in neuronal differentiation, and several aspects of neurite outgrowth, including elongation, calibre and branching, are regulated by activated Akt [59]. These findings suggest that IMR-32 neuroblastoma cells after treatment with ASH-WEX with relatively high P-Akt levels may remain well-differentiated and exhibit a slower growth rate. P-Akt post translational change is the activated form of Akt and changes in expression of P-Akt at protein level and Akt at mRNA level could be due to post-translational modifications for signalling mechanisms. We propose that the increase in Akt-P expression on treatment with ASH-WEX is an indicator of induction of cell differentiation in the IMR-32 cells.

NCAM, is required for neurite outgrowth in culture and in axonal guidance and setting up of the neuronal network *in vivo* [60], [61]. NCAM is a developmentally regulated protein and is implicated in a variety of cellular processes, such as cell-cell adhesion, cell migration, neurite outgrowth, and synaptic plasticity. The present data shows highly up-regulated expression of NCAM upon 0.5% ASH-WEX treatment. Interestingly, low NCAM expression has been shown to relate with clinically aggressive cancers and vice-versa [62]. In the present study, NCAM was highly expressed in ASH-WEX treated group and also appeared to be translocated into the growing neurites that develops upon differentiation, thus suggesting their participation in neurite outgrowth and adhesion. These results are in line with our previous study in which ASH-WEX has been shown to induce NCAM expression in glioma cells [29]. NCAM undergoes post-translational modification including the addition of PSA chains on its extracellular domain [63]. Expression of PSA is associated with cellular migration, axon induction and also contacts with their target [64]. PSA expression in most cancer cells is correlated with tumor metastasis and associated with tumor differentiation as well as serves as an onco-developmental antigen [65], [66]. In the present study, ASH-WEX significantly reduced the surface expression of PSA-NCAM and these findings are also supported by Western blotting results. ASH-WEX treatment seems to downregulate polysialylation through inhibition of PST enzyme as suggested by RT-PCR results of PST expression.

The decrease in surface expression of PSA-NCAM may be attributed to differentiated phenotype of IMR-32 cells. Neuroblastoma proliferation has been shown to be facilitated by polysialylation of NCAM and surface expression of PSA is regulated at the level of polysialyltransferase transcription [67]. PSA expression in cancerous cells appears to facilitate mitosis and metastasis [68]. Bertozzi’s group showed that feeding cells with N-butylmannosamine can inhibit polysialyltransferase activity and suggested that glycosylation efficiency may decrease substantially when the structure of a precursor carbohydrate residue is modified [69], [70]. Regulation of polysialyltransferase expression was suggested to occur at the transcription-initiation level [71], [72]. Nakagawa *et al.* [73] demonstrated that the cAMP-CREB pathway regulates polysialyltransferase expression in cell culture. In addition, Bruses and Rutishauser [74] have proposed a calcium-dependent regulatory mechanism for enzyme activity. It has also been reported that polysialyltransferase phosphorylation may be involved in regulation of PSA expression [66]. Thus present findings compare well with previous results on other PSA-expressing tumors [65], in which polysialyltransferase mRNA and PSA expression correlates with tumor progression.

To further assess the anti-migratory potential role of ASH-WEX in neuroblastoma, MMPs expression was studied by gel zymography. MMPs play an important role in tumor invasion and metastasis [75], [76]. MMP-2 and MMP-9 appear to be differentially expressed during development of the rat CNS [77]. It has been known that increase in the expression, secretion in the media, and activation of MMP-2 and 9, leads to a more tumorigenic phenotype due to increased MMP-2 mediated invasion [78], [79]. It is also well known that metastatic aggressiveness of the tumor is inversely related to its differentiation status. Earlier studies have established that overexpression of cyclin D1 leads to an increase in invasive properties of cells and cyclin-D1 expression was associated with the increased gelatinolytic activity of proMMP-2 and MMP-2 [80]. ASH-WEX treatment was observed to downregulate Cyclin D1 expression as well as activity/expression of MMP2 and 9 thus indicating its anti-invasive/migratory properties.

Previous studies have established that the active components of ASH-WEX are neither heat labile, nor proteinaecious and nucleic acid in nature [29] which needs to be further investigated. In the present study, ASH-WEX induced upregulation of NF200, HSP70 and mortalin expression may be correlated with the induction of differentiation in these neuroblastoma cells which was even better than the RA treatment groups. The upregulation of NCAM and downregulation of PSA-NCAM and MMPs may explain the anti-migratory and differentiation inducing properties of ASH-WEX. The decrease in Cyclin D1 and bcl-xl expression and enhanced expression of Akt-P may be resulting in arrest of IMR-32 cell proliferation and their differentiation into mature neuron-like cells. FACS analysis demonstrated that ASH-WEX caused an arrest of the cell cycle in the G0/G1 phase with a decrease of cell population in synthesis and mitosis phases in IMR-32 cells. Since most of the antineoplastic drugs in clinical use block the cell cycle in the S or G2/M phases, whereas, ASH-WEX blocks the cell cycle in the G1 phase, a combination of ASH-WEX with currently used drugs might possibly improve therapies of neuroblastoma. Overall ASH-WEX treatment shows decreased neuroblastoma cell proliferation, cell migration in addition to induction of senescence and cell cycle arrest leading to differentiated phenotype. ASH-WEX appears to affect multiple pathways for its anti-cancer and differentiation inducing role in IMR-32 neuroblastoma cells instead of targeting a single protein or pathway which needs to be further studied. The current study supports the idea that ASH-WEX may have the potential to reduce the malignancy of neuroblastomas. Low dose efficacy of ASH-WEX with its differentiation inducing potential may suggest that it could be a potential candidate for adjunct therapy of neuroblastomas and glioblastomas.

## Supporting Information

Figure S1 To ascertain the effect (if any) of the vehicle, DMSO in the RA treated group, DMSO (1 µl/ml) treated cells were studied along with the control (untreated) IMR-32 cells for 72 hrs. DMSO was not found to affect the cell morphology (as indicated by phase contrast photographs) and expression of NF200 and HSP70 in these cultures when compared with untreated control cells. DAPI stain was used as counterstain to visualize the nucleus.
(TIF)
