# Supplementary figures and images for: Withania somnifera Water Extract as a Potential Candidate for Differentiation Based Therapy of Human Neuroblastomas
Source: PLoS One. 2013 Jan 31;8(1):e55316. doi: 10.1371/journal.pone.0055316 (PMC3561198; doi:10.1371/journal.pone.0055316)

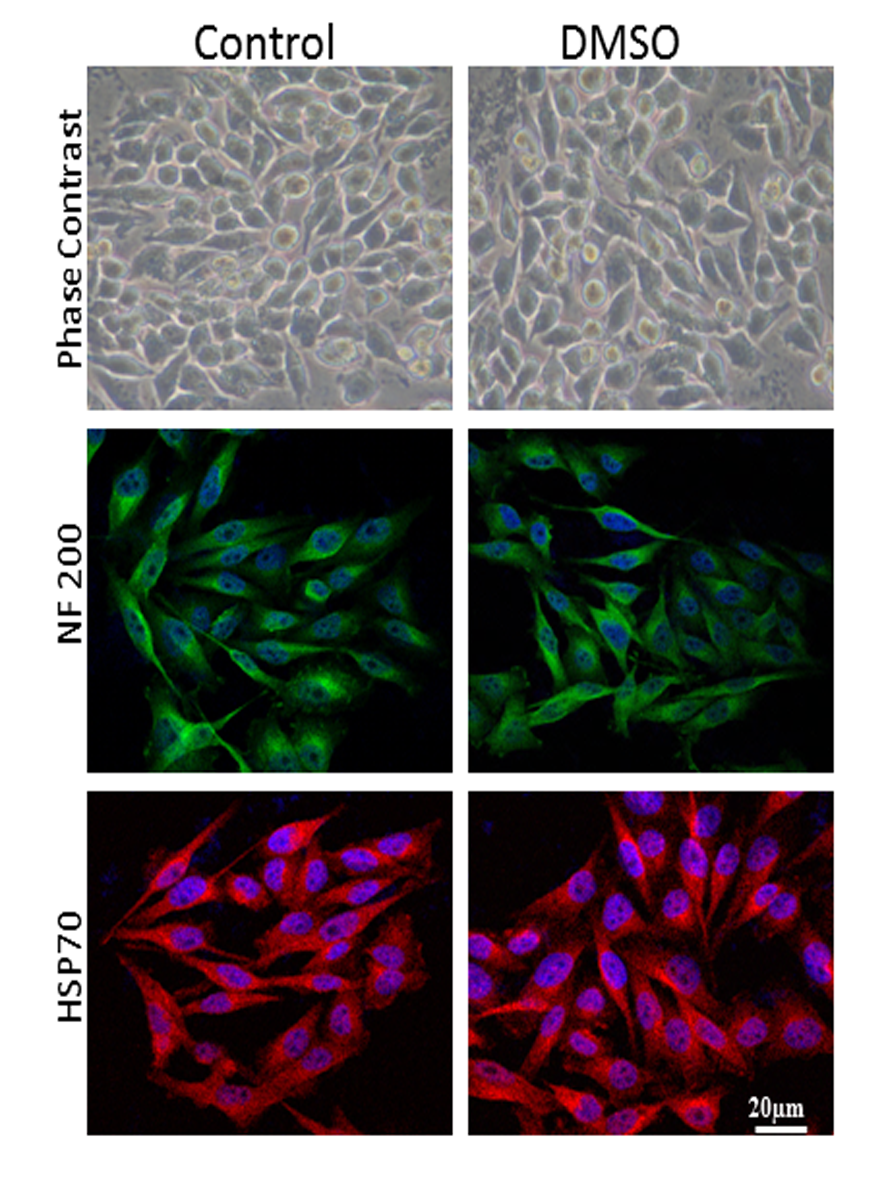

Supplement: Figure S1 — To ascertain the effect (if any) of the vehicle, DMSO in the RA treated group, DMSO (1 µl/ml) treated cells were studied along with the control (untreated) IMR-32 cells for 72 hrs. DMSO was not found to affect the cell morphology (as indicated by phase contrast photographs) and expression of NF200 and HSP70 in these cultures when compared with untreated control cells. DAPI stain was used as counterstain to visualize the nucleus. (TIF) [file pone.0055316.s001.tif]
